# Supplementary material for: The genome sequence of the Scarce Vapourer, Orgyia recens (Hübner, 1819) (Lepidoptera: Erebidae)
Source: Wellcome Open Res. 2025 Oct 21;10:590. [Version 1] doi: 10.12688/wellcomeopenres.25063.1 (PMC12831957; doi:10.12688/wellcomeopenres.25063.1)
Supplement: Peer review history

# Reviewer report: Reviewer response for version 1

**DOI:** 10.21956/wellcomeopenres.27629.r144410

**Published:** 2026-01-24

**Authors:** Peter Huemer

**Affiliations:** 1 Tyrolean Federal State Museums, Innsbruck, Austria

A well-written data note, which, however, from the reviewer’s perspective, contains a somewhat geographically unbalanced background description of the species. References from continental Europe would be very helpful here, for example:

- Reference to additional larval host plants such as *Betula* spp. (Ebert 1994)
- High level of threat and inclusion in Red Lists (Ebert 1994, Huemer 2007)

**Specific remark:**

 “Larvae are present from July onwards. They overwinter as larvae in a loose silk spinning between two leaves of the food plant, resume feeding in April, and pupate in May.”

 This sentence describes only the first generation.

 In addition, due to the risk of confusion for this species, it would seem sensible to include a note on morphological differences compared with the widely distributed European species *Orgyia antiqua.*

 Ebert, G. (ed.) (1994): Die Schmetterlinge Baden-Württembergs. Band 4: Nachtfalter II. Eugen Ulmer, Stuttgart, 536 pp.

 Huemer P. (2007): Rote Liste ausgewählter Nachtfalter Österreichs (Lepidoptera: Hepialoidea, Cossoidea, Zygaenoidea, Thyridoidea, Lasiocampoidea, Bombycoidea, Drepanoidea, Noctuoidea), pp. 199‒361. – In: ZULKA K.P. (Red.): Rote Listen gefährdeter Tiere Österreichs. Checklisten, Gefährdungsanalysen, Handlungsbedarf. Teil 2: Kriechtiere, Lurche, Fische, Nachtfalter, Weichtiere. Grüne Reihe des Lebensministeriums 14/2, 515 pp.

Are sufficient details of methods and materials provided to allow replication by others?

Yes

Is the rationale for creating the dataset(s) clearly described?

Yes

Are the datasets clearly presented in a useable and accessible format?

Yes

Are the protocols appropriate and is the work technically sound?

Yes

Reviewer Expertise:

Integrative taxonmic expertise in European Lepidoptera

I confirm that I have read this submission and believe that I have an appropriate level of expertise to confirm that it is of an acceptable scientific standard.

# Reviewer report: Reviewer response for version 1

**DOI:** 10.21956/wellcomeopenres.27629.r136934

**Published:** 2025-11-22

**Authors:** Josephine R Paris

**Affiliations:** 1 University of Exeter Library, Devon, UK

This a good Data Note, which contains good background information for the species (the Scarce Vapourer). The methods contain a thorough description of the library preparation and bioinformatics methods. The genome quality is very good and it's nice that both sex chromosomes were sequenced and assembled.

 Small comments to improve readability:

 It seems as though commas have been replaced by a space in all numbers in the thousands, e.g. in the abstract describing the number of protein-coding genes identified in the abstract: 10 917 and in the description of the Genome annotation report. This may be a rendering issue, but fixing this would be appreciated to improve readability.

 In the background section, my preference would be to present the species' overall distribution first, i.e. "Its wider range extends from the north of the Iberian Peninsula east across western and central Europe into East Asia (including Japan), north to southern Scandinavia and south to the Mediterranean ( The Butterfly Foundation, 2025)." before making specific references to it's restricted distribution in Great Britain (leading it to be listed as a Red Data Book species).

 Datasets are available via the links given in the text.

Are sufficient details of methods and materials provided to allow replication by others?

Yes

Is the rationale for creating the dataset(s) clearly described?

Yes

Are the datasets clearly presented in a useable and accessible format?

Yes

Are the protocols appropriate and is the work technically sound?

Yes

Reviewer Expertise:

population genomics of non-models, genome assembly and annotation

I confirm that I have read this submission and believe that I have an appropriate level of expertise to confirm that it is of an acceptable scientific standard.
